# Giant parosteal lipoma of thigh: A case report and review of the literature

**DOI:** 10.1016/j.ijscr.2025.111273

**Published:** 2025-04-16

**Authors:** Huy Hoang Quoc, Vinh Pham Quang, Bach Nguyen, Binh Le Nguyen, Son Do Le Hoang

**Affiliations:** a Department of Orthopaedics and Rehabilitation, University of Medicine and Pharmacy at Ho Chi Minh City, 217 Hong Bang Street, District 5, Ho Chi Minh City, Viet Nam; b Department of Orthopaedics, University Medical Center Ho Chi Minh City, 201 Nguyen Chi Thanh Street, District 5, Ho Chi Minh City, Viet Nam; c Department of Orthopaedic, Cho Ray Hospital, 201 Nguyen Chi Thanh Street, District 5, Ho Chi Minh City, Viet Nam

**Keywords:** Lipoma, Periosteum, Margins of excision

## Abstract

**Introduction and importance:**

Parosteal lipoma is an uncommon benign tumor that can arise in various parts of the body. Giant parosteal lipomas are particularly rare and may compress or interfere with adjacent structures. Effective management typically involves surgical removal, with a thorough evaluation to exclude the possibility of malignancy.

**Case presentation:**

We describe a case of a patient presenting with a soft tissue mass in the thigh measuring 10 cm × 14 cm × 26 cm and weighing 1300 g. MRI findings were suggestive of a parosteal lipoma. Wide excision of the tumor was performed using an anterior thigh approach. Histopathological examination confirmed the diagnosis of parosteal lipoma. The postoperative course was uneventful, with no complications or local recurrence observed.

**Clinical discussion:**

Most lipomas are asymptomatic; however, in some cases, they can affect nearby structures, potentially leading to compression syndromes. MRI is the preferred imaging technique for evaluating large soft tissue tumors with suspected malignancy. It plays a crucial role in distinguishing between differential diagnoses and facilitating treatment planning. Surgery is recommended for tumors that are large, symptomatic, compress vital structures like nerves or blood vessels, or are suspected to be malignant. Performing a wide tumor excision is crucial to reduce the likelihood of recurrence.

**Conclusion:**

Malignancy should be carefully ruled out in diagnosing giant parosteal lipoma. MRI plays a crucial role in distinguishing between potential diagnoses, and aiding in surgical planning. Complete tumor excision through wide resection is the treatment of choice for this lesion.

## Highlights

• Giant parosteal lipoma is a rare lipomatous lesion that can be symptomatic when compressing surrounding structures.
• MRI was crucial for diagnosis, distinguishing it from malignancies like liposarcomas
• Wide excision is needed to minimize recurrence in large symptomatic tumors

## 1 Introduction

Parosteal lipoma is a rare subtype of lipomatous lesion, accounting for about 0.3 % of all lipomas. It localized mostly in the thigh, forearm, calf, and arm, and this lesion typically occurs in middle-aged individuals, from 40 to 60 years old [1]. Lipomas can develop on any part of the body, but they most commonly occur on the trunk and upper extremities. Typically small and soft, they can occasionally be relatively firm and grow to a notable size. Giant lipomas are characterized as those measuring at least 10 cm in one dimension or weighing a minimum of 1000 g, and lesions in the femur tend to be larger than those at other sites [2]. Surgical resection is the treatment of choice for this lesion. However, because bony lesions is often found in 59.2 % of parosteal lipoma cases [3], it is crucial to consider malignant tumors in the differential diagnosis. This study is aiming to review the diagnosis procedure and treatment method in giant lipoma with bony lesion. The case report is reported in line with SCARE criteria [4].

## 2 Case presentation

**Fig. 1:**
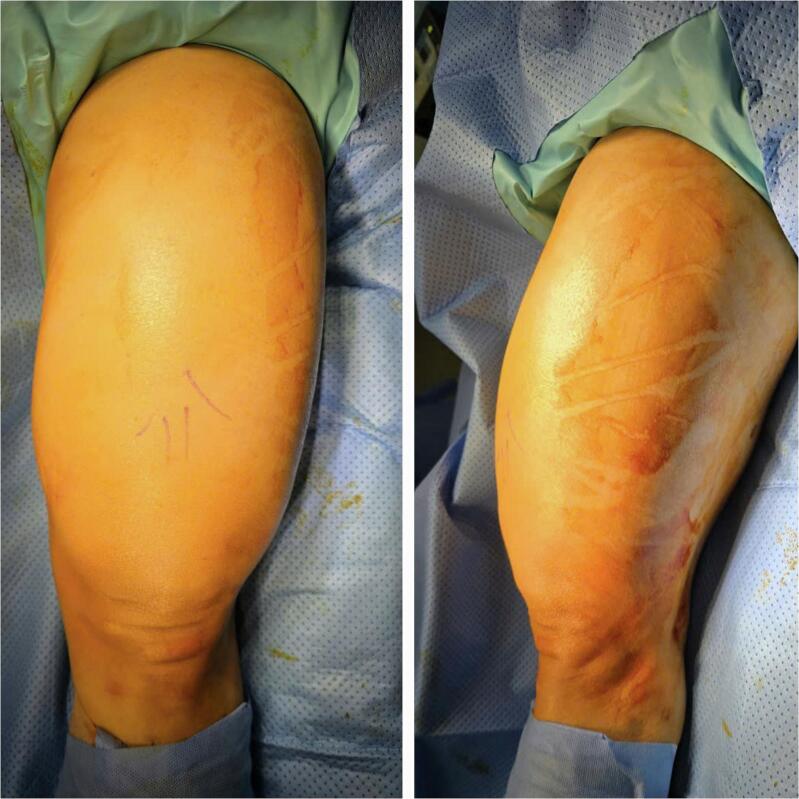
Clinical appearance of giant mass in left thigh.

**Fig. 2:**
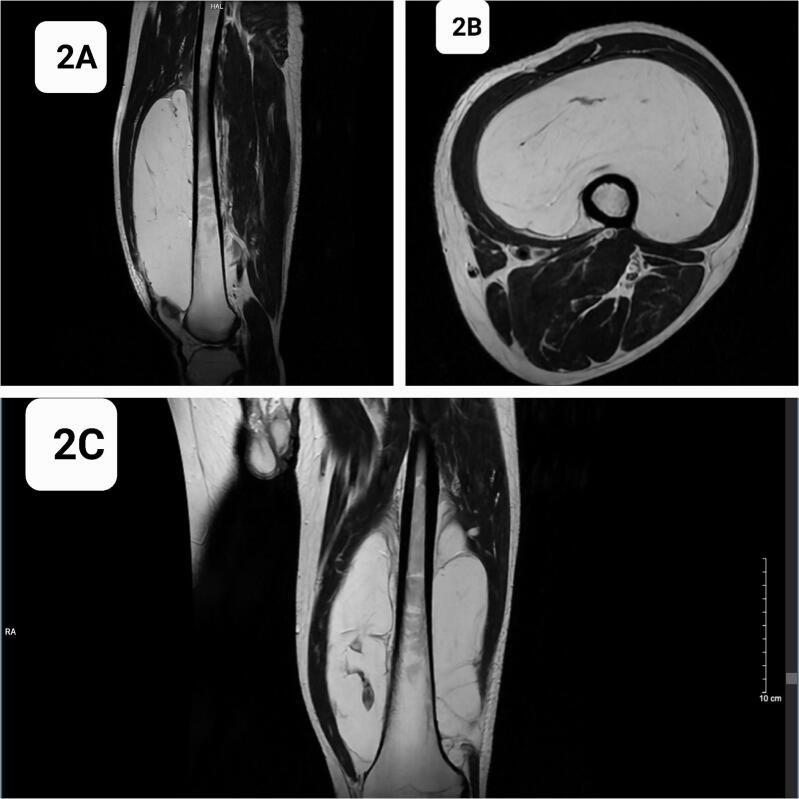
Giant lipomatous tumor around left femur on T2-weighted sequences MRI.

A 54-year-old male with a medical history of diabetes and hypertension presented with a progressively enlarging mass in the anterior aspect of the left thigh. The mass had been present for approximately 10 years, with a slow rate of growth. Recently, the lesion became symptomatic, causing heaviness in the lower extremity during ambulation and difficult with clothing due to its size. The patient denied associated symptoms, including pain, paresthesia, pruritus, or discharge from the lesion. There was no history of prior trauma, surgical intervention, or exposure to radiation in the affected region. Physical examination revealed a large, dome-shaped, firm mass measuring approximately 24 cm × 15 cm, occupying nearly the entire length of the anterior thigh (Fig. 1). The mass was nontender and non-pulsatile, with no evidence of overlying skin changes or regional lymphadenopathy. Distal neurovascular examination of the affected limb was within normal limits. Magnetic resonance imaging demonstrated a large, well-circumscribed lesion measuring 90 mm × 150 mm × 215 mm originating from the anterior surface of the femur. The lesion extended from the proximal femoral region near the inguinal ligament to the anterior aspect of the bilateral femoral condyles, occupying the majority of the anterior thigh compartment. The mass displaced adjacent musculature without evidence of infiltration. Signal characteristics included homogeneous hyperintensity on both T1- and T2-weighted sequences, with focal areas of internal calcification. There was no evidence of peri-lesional vascular proliferation, cortical bone erosion, or marrow invasion (Fig. 2). These imaging findings were consistent with a diagnosis of parosteal lipoma. (See Table 1.)

**Table 1:** Differential diagnosis of parosteal lipoma based on MRI with contrast.

Condition	Key MRI Features	Contrast Enhancement
Parosteal Lipoma	Primarily fat signal (T1 hyperintense, T2 hyperintense, suppressed on fat-sat). Minimal to no enhancement. May show mild enhancement if fibrous or osseous components are present.	Minimal to none
Well-Differentiated Liposarcoma	Contains fat but with thickened fibrous septa (>2 mm) and nodular enhancing soft tissue components. Moderate to intense enhancement in non-fatty regions. Infiltrative borders.	Moderate to intense in non-fatty areas
Parosteal (Juxtacortical) Osteosarcoma	Low T1, high T2 signal with variable contrast enhancement. Cloud-like or amorphous bone formation. Cortical invasion and soft tissue extension.	Variable, may be intense
Periosteal Chondrosarcoma	Lobulated, cartilage-rich tumor with high T2 signal. Ring-and-arc enhancement pattern typical of chondroid tumors. Often associated with cortical scalloping or bone destruction.	Ring-and-arc pattern
Osteochondroma	Cortical continuity with parent bone. Cartilage cap shows mild to moderate enhancement. No significant enhancement of the bony stalk.	Mild to moderate in cartilage cap

**Fig. 3:**
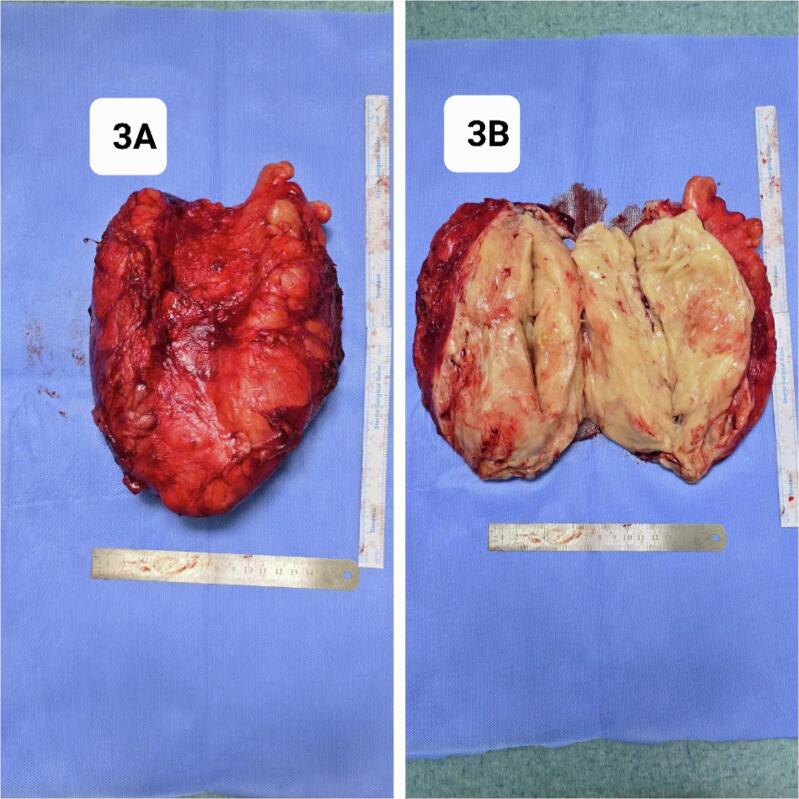
Excised parosteal lipoma.

**Fig. 4:**
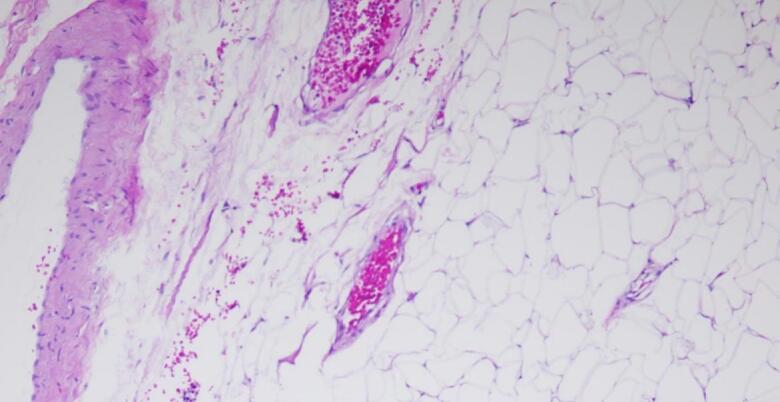
Histology of benign lipoma.

After discussing the patient's condition and treatment options, surgical excision was agreed upon with the patient's informed consent. The surgery was performed under spinal anesthesia with the patient in the supine position. A midline longitudinal incision was made along the anterior aspect of the left thigh. The rectus femoris muscle group was carefully dissected to expose the tumor. The tumor capsule was preserved intact, and the mass was completely excised down to the periosteum of the femur while preserving the surrounding thigh musculature. The excised tumor measured 10 cm × 14 cm × 26 cm and weighed 1300 g (Fig. 3). Histology result was benign lipoma (Fig. 4).

The postoperative recovery was uneventful. The surgical drain was removed after two days, and the patient was discharged three days postoperatively. The patient resumed normal ambulation within one week. Histological examination revealed a benign adipocytic tumor consistent with a parosteal lipoma. The tumor predominantly comprised mature adipocytes, with focal areas of calcification. These findings confirmed the preoperative diagnosis of parosteal lipoma.

## 3 Discussion

Lipomas are the most prevalent soft tissue tumors, accounting for nearly half of all benign mesenchymal tumors. A lipoma is a benign growth made up of mature adipose tissue, indistinguishable histologically from normal fat [5]. 

Parosteal lipoma, first identified by Dr. Seerig in 1836, is a rare benign neoplasm arising from mature adipose tissue. The term “parosteal lipoma,” popularized by D'Arcy Power in 1888, highlights the tumor's relationship with the bone. The term “parosteal lipoma” is preferred over “periosteal lipoma” because periosteal tissue lacks fat cells. Parosteal lipomas account for only 0.3 % of all lipomas and 0.1 % of primary bone neoplasms [6]. The most common sites for parosteal lipomas include the femur, proximal radius, humerus, tibia, clavicle, and pelvis. Although rare, they can also occur in the ribs [7].

Parosteal lipomas, which contain fat tissue along with regions of bone and cartilage differentiation, are generally situated within subcutaneous fat or muscle. Symptoms can vary depending on their location. Diagnosis typically relies on a combination of patient history, physical examination, and further diagnostic evaluations. Most patients present with a painless mass, without any motor or sensory deficits, and lymph node assessments usually show no abnormalities. However, in rare cases, they can cause compression syndromes due to nerve damage and difficulties in walking. Therefore, clinical diagnosis can sometimes be challenging in certain cases [8].

Lipomas exhibit distinct imaging characteristics. On ultrasound, lipomas present as hyperechoic lesions, similar in echotexture to muscle tissue. Ultrasound is often used as an initial approach for evaluating a “giant lipoma.” However, its reliability may be limited, as the diagnostic outcome heavily depends on the radiologist experience [9]. On radiographic imaging, parosteal lipomas appear as well-defined radiolucent masses resembling periosteal reactions. Periosteal responses around the tumor may include periosteal thickening, calcification, or fibrosis. Smooth scalloping of the cortex, osseous bowing, or projection can be observed [10]. On CT scans, these lesions appear as well-defined, low-density masses (−120 to −60 Hounsfield units) that do not enhance with contrast administration. MRI is a highly effective diagnostic tool, particularly in cases where malignant lesions such as liposarcoma are suspected. On MRI, parosteal lipomas exhibit high signal intensity on T1- and T2-weighted images and signal loss on fat-suppression sequences, without enhancement with contrast, similar to subcutaneous fat. MRI particularly effective in detecting muscle atrophy and assessing nerve compression or involvement when present. The most critical differentiation is between benign lipomas and well-differentiated liposarcomas. Benign lipomas typically present as masses with regular margins, except for cases of infiltrating lipomas, which may deviate from this pattern. Most benign lipomas demonstrate homogenous fat signal on MRI or fibrous components. Well-differentiated liposarcomas, on the other hand, often display septations or lobulations with heterogeneous signals of non-adipose areas. These septations or lobulations exhibit greater enhancement on post-contrast MRI compared to benign lipomas [11]. Some features suggestive of well-differentiated liposarcoma include a size >10 cm, fat content below 75 %, and the presence of thick septa >2 mm [12].

The cytogenetic and molecular characteristics of parosteal lipomas remain incompletely characterized; however, they exhibit genetic alterations consistent with those observed in soft tissue lipomas, suggesting a shared pathogenesis. Approximately 60 % of soft tissue lipomas demonstrate karyotypic abnormalities, with chromosomal translocations involving 12q13-q15 being the most prevalent. Among these, chromosome 3q27-q28 is the most frequent translocation partner, occurring in up to 25 % of cases involving 12q13-q15 [13]. The principal genes implicated in this translocation include HMGI-C (HMGA2) at 12q15 and LPP at 3q27–28, both of which play roles in adipocytic tumorigenesis. Furthermore, various other chromosomes have been identified as translocation partners with chromosome 12 in soft tissue lipomas. Notably, parosteal lipomas have been found to harbor the t(3;12) translocation, reinforcing the hypothesis of a common molecular etiology with soft tissue lipomas [14]. Ka'roly Szuhai et al. concluded that parosteal liposarcoma is not a separate entity but should be categorized within the spectrum of soft-tissue ALT/WDLS [15].

The treatment options for parosteal lipomas include surgical intervention or conservative management. The choice of treatment depends on various factors such as tumor size, clinical symptoms, suspicion of malignancy, comorbidities, and patient preferences. Surgical biopsy could be indicated for firm, rapidly enlarging lipomatous masses [16]. However, concerns remain regarding the risks of recurrence [17], hematoma formation, or the potential for well-differentiated liposarcoma [18].

Complete surgical excision of parosteal lipomas is essential to prevent recurrence. Surgical approach should allow for the complete removal of the tumor while preserving vital adjacent structures such as nerves and blood vessels. The surgical objective is a wide excision of the tumor often feasible since parosteal lipomas typically exhibit a pseudocapsule and well-defined boundaries from surrounding tissues. In our case, we use anterior direct approach, making a longitudinal incision through the vastus intermedius muscle after dissecting between the rectus femoris and vastus lateralis plane. The advantage of this approach lies in its ability to fully access the tumor along its entire length, from the hip joint to the knee region, and to address both the medial and lateral aspects of the tumor. McRae has described an anterolateral approach between the vastus lateralis and vastus intermedius to access the femoral shaft. Additionally, another approach between the vastus lateralis and the intermuscular septum can also provide access to the femur, but it poses challenges in reaching anterior-medial tumors. Proximally, there is a risk of injury to the superior medial geniculate artery, the nerve to the vastus lateralis, and the lateral femoral circumflex artery if the approach extends near the hip joint. Distally, critical structures such as the popliteal artery and sciatic nerve must be preserved.

The main drawback of anterior approaches is the risk of postoperative quadriceps adhesions. Removal of a large tumor carries the risks of thrombus formation and dead space creation [19]. Thorough intraoperative hemostasis, the use of drains, and postoperative compression bandaging help minimize the formation of dead space.

Local recurrence can occur even after wide excision, post operative follow-up is important to early detection of local recurrence [20].

## 4 Conclusion

Giant parosteal lipomas are rare. Soft tissue tumor larger than 10 cm or weighing >1 kg, the possibility of malignancy must be excluded. The most critical differential diagnosis is well-differentiated liposarcoma. MRI is a valuable tool for differentiating diagnoses, assessing the tumor's relationship with surrounding structures, and planning surgical intervention. Wide excision surgery allows for complete tumor removal, reducing the risk of local recurrence.

## CRediT authorship contribution statement

*Vinh Pham Quang:* conceptualising the plan for surgery, performing the surgery, follow up patient's recovery, reviewing the manuscript.

*Huy Hoang Quoc:* Assisting in planning and in the surgery, writing the draft for case report, writing the literature review for case report.

*Bach Nguyen:* Assisting the surgery, writing the literature review, taking note of postoperative function.

*Binh Le Nguyen:* taking note and data visualisation perioperatively.

*Son Hoang Do Le:* Assisting the surgery, prepare the necessary equipments.

## Consent

Written informed consent was obtained from the patient for publication of this case report and accompanying images. A copy of the written consent is available for review by the Editor-in-Chief of this journal on request.

## Ethical approval

This study in our institution was in normal procedure with surgery consent form. The Ethical approval is not needed for case report according to Ethical Committee of University Medical Center.

## Sources of funding

None.

## Declaration of competing interest

All authors declare no financial or personal relationship with other entities that could inappropriately influence this study.
